# Urban vs rural – Prevalence of self-reported allergies in various occupational and regional settings

**DOI:** 10.1016/j.waojou.2022.100625

**Published:** 2022-01-27

**Authors:** Linda Tizek, Elisa Redlinger, Johannes Ring, Kilian Eyerich, Tilo Biedermann, Alexander Zink

**Affiliations:** a Technical University of Munich, School of Medicine, Department of Dermatology and Allergy, Biedersteinerstraße 29, Munich, 80802, Germany; b Division of Dermatology and Venereology, Department of Medicine Solna, Karolinska Institutet, Stockholm, Sweden

**Keywords:** AIT, allergen specific immunotherapy, CI, confidence interval, ENT, ear nose throat specialist, GP, general practitioner, OR, odds ratio, OTC, over-the-counter, SD, standard deviation, ZLF, Bavarian Central Agricultural Festival, Allergy, Prevalence, Rural, Treatment, Urban

## Abstract

**Background:**

Allergies have an enormous individual and economic impact worldwide and affect more than one quarter of the population in Germany. Various factors influence the development of allergies: besides genetic predisposition the environment in which a person is raised and living also plays a role. The aim of the study was to evaluate differences in allergy prevalence in relation to age, sex, occupation, and living area (settlement structures).

**Methods:**

A cross-sectional study using a paper-based questionnaire about allergies was performed at the Munich Oktoberfest 2016. Participants were divided into 4 occupational groups and compared using descriptive statistics and multiple regression.

**Results:**

Overall, 2701 individuals (mean age 51.9 ± 15.3 years; 53.5% women) participated in the study. The overall rate of any self-reported allergy was 27.3% in the study population, in which women were more likely to be affected than men (OR = 1.82; 95% CI [1.50; 2.22]). Compared to farmers, all other occupational groups had a higher risk of reporting pollen allergies. Participants from rural areas (OR = 0.38; 95% CI [0.26; 0.58]) and suburban areas (OR = 0.44; 95% CI [0.30; 0.64]) were significantly less affected by allergies than participants from urban areas. Around 45.2% of the participants affected by allergies reported not receiving any treatment at all.

**Conclusion:**

Differences in the self-reported prevalence of allergies were shown for age groups, sex, living area, and occupation. Especially the reported pollen allergy prevalence ranged widely between different occupations, indicating that those individuals with an occupational exposure to pollen may have a lower risk than indoor workers. Overall, there remains a high need for sufficient treatment of allergies.

## Introduction

A large proportion of the global population is affected by allergies with a considerable impact on affected individuals and the communities in which they live.^1^ In Germany, the prevalence of allergies was shown to be between 20 and 40%^2^, ^3^, ^4^, ^5^, ^6^, ^7^ depending on age, sex, settlement structure, and social status.^7^^,^^8^ Common allergic symptoms include rhinitis, rhinosinusitis, conjunctivitis, allergic asthma, and urticaria, and in severe cases, can manifest themselves as life-threatening anaphylaxis.^5^ As a result, the quality of life of affected individuals can be impaired severely, particularly with regard to school and academic performance.^9^^,^^10^

Previous studies demonstrated the protective effect of some factors on allergy development.^11^, ^12^, ^13^, ^14^ For example, growing up on a farm or having a mother who worked on a farm during pregnancy was shown to have a protective effect on the development of allergic diseases in childhood through adulthood.^11^^,^^12^ This can be explained by the exposure to farm dust, which contains endotoxins shown to modify immune responses through enzyme A20 induction and therefore could reduce the risk of developing allergies.^13^ This exposure to bacterial and viral components is part of the "hygiene hypothesis", which states that decreased stimulation of the immune system may contribute to the increased prevalence of allergic diseases.^14^ Additionally, people living in rural areas often have outdoor occupations like farming and foresting, while people living in urban areas often work indoors in scientific or technical services.^15^ These different occupational patterns could influence the development of allergies because of varying degrees of allergen exposure. For example, the exposure to grass pollen is especially high during feeding sessions on farms.^16^ Furthermore, regarding bee and wasp venom allergies, it was shown that sensitization levels and anaphylaxis risk rises with the quantity of stings, which is important for outdoor workers like fishers and hunters, who have a higher risk of being stung.^17^

Hymenoptera venom allergies and respiratory allergies can be treated by allergen specific immunotherapy (AIT), which not only reduces allergic symptoms but could also prevent other sensitisations and the development of asthma.^18^ Additionally, AIT is a new approach for treating people affected by food allergies and at risk for anaphylaxis after accidental exposure.^5^^,^^19^ Despite different means such as pharmacotherapy, immunotherapy, and environmental control to reduce allergy symptoms,^5^ a high proportion of people with allergies stated that their allergy had a negative impact on their daily life and that treatment was not sufficient.^4^ Only about two-thirds of affected people are treated by physicians^3^ and only about one-third of people with pollen allergies receive AIT.^4^

Accordingly, the aims of this study were to assess the self-reported point prevalence of allergies and to identify possible associations with age, sex, living environment, and occupation. Furthermore, we investigated the differences in utilization of treatment modalities for allergies.

## Methods

A cross-sectional study was performed among visitors of the 2016 “Bavarian Central Agricultural Festival” (ZLF, Bayerisches Zentral-Landwirtschaftsfest), which takes place every 4 years as part of the Munich Oktoberfest.^20^ This unconventional setting was chosen to include people who usually do not consult a physician regarding their allergies. Visitors were asked to fill out a self-administered questionnaire, which included questions on sex, age, postal code, and occupation. People were asked whether they had any allergic disease and — if so — to specify against what allergen (pollen, house dust mite, food, animal, contact, drug, insect, and other allergies with a possibility to specify using a free-text field). If they indicated having at least one allergy, they were also asked how their allergies are treated (no treatment, treatment by a general practitioner (GP), dermatologist, or ear nose throat specialist (ENT), self-treatment with or without over-the-counter (OTC) drugs, treatment by a non-medical practitioner, and “other”). The study team was available for support for questions that arose during the questionnaire. For study participation, all participants had to be at least 18 years old and had to provide written informed consent prior to study inclusion. The study was approved by the ethics committee of the Medical Faculty (Reference 385/16 s).

To assess differences within the population, participants were classified into 5 age groups: 18–39 years, 40–49 years, 50–59 years, 60–69 years, and 70+ years.^21^ Participants were then assigned into four occupational groups: (i) farmers in main occupation, (ii) other outdoor occupations (eg, gardener, forester, construction worker), (iii) indoor occupations (eg, office worker, medical technician, mechatronics engineer), and (iv) other occupations (e.g. both office worker and farmer as a secondary occupation), which could not be assigned to the other groups because they included indoor and outdoor work. Pensioners who did not give details about their former occupation were not included in the occupational groups sub-analysis.

Using the provided postal codes, 3 categories were derived for different living areas in Bavaria as follows: "urban areas", "suburban areas", and "rural areas". (i) Urban areas included cities with at least 100 000 inhabitants; (ii) suburban areas were defined as areas with a population density of at least 150 inhabitants/km^2^ or having at least 50% of the inhabitants living in medium-sized towns between 20 000 and 99 999 inhabitants; (iii) rural areas were defined as sparsely populated areas in which less than 50% of the inhabitants are living in medium-sized towns or which have a population density less than 100 inhabitants/km^2^.^22^

### Statistical analysis

Data were analysed using descriptive statistics. The Student's t-test was used to detect age differences between sex, a one-way analysis of variance (ANOVA) was used to detect age differences between living areas and occupational groups, and Pearson's Chi square or Fisher Exact test were used to detect differences for categorical variables. Univariate and multivariate logistic regressions using backward selection were applied with dependent variables set as the presence of any allergy and as the different categories of allergies (pollen, house dust mite, food, animal, and contact). Sex, age group, occupation, and the area type were used as independent variables. For the analysis of treatment, the kind of treatment was defined as a dependent variable by applying multivariate logistic regression using backward selection. Like in the allergy analysis, sex, age group, occupation, and the area type were chosen as independent variables.

*P*-values <0.05 were considered statistically significantly and 95% confidence intervals (CI) were generated. To analyse the prevalence of allergies in the total population and within the different Bavarian areas (n ≥ 15 participants), 1000 samples bootstrapping with 95% CI was conducted. IBM SPSS Statistics 26 (IBM Corporation, Armonk, NY, USA) was used for all analyses.

## Results

Overall, 2701 individuals (53.5% women) with a mean age of 51.9 ± 15.3 years (range 18–90 years) participated in the study. Most participants lived in suburban areas (63.9%), whereas 27.0% lived in rural areas and 6.0% lived in urban areas. The distribution of farmers (36.1%) and indoor workers (32.5%) within the cohort was almost balanced.

The proportion of men was considerable lower among indoor workers (29.1%) compared to all other occupational groups. Indoor workers (mean age 45.2 ± 16.4 years) were significantly younger than all other occupational groups, and the proportion of indoor workers was higher in urban areas (86.2%) than in suburban areas (32.6%) and rural areas (31.3%, Table 1).

**Table 1:** Baseline characteristics of the study population separated by occupational groups.

	All (n = 2701) n (%)	Farmers (n = 975; 36.1%) n (%)	Other outdoor workers (n = 415; 15.4%) n (%)	Indoor workers (n = 879; 32.5%) n (%)	Other occupations (n = 217; 8.0%) n (%)
**Age in years (mean ± SD)**	51.9 ± 15.3	56.4 ± 12.5	54.5 ± 14.1	45.2 ± 16.4	50.4 ± 13.7
Missing	63 (2.3%)	20 (2.3%)	11 (2.7%)	16 (1.8%)	4 (1.8%)
**Sex**					
Men	1248 (46.2%)	522 (53.5%)	250 (60.2%)	256 (29.1%)	124 (57.1%)
Women	1445 (53.5%)	452 (46.4%)	165 (39.8%)	623 (70.9%)	93 (42.9%)
Missing	8 (0.3%)	1 (0.1%)	0	0	0
**Settlement structure**					
Urban area	161 (6.0%)	3 (0.3%)	6 (1.4%)	112 (12.7%)	9 (4.1%)
Suburban area	1726 (63.9%)	665 (68.2%)	273 (65.8%)	526 (59.8%)	148 (68.2%)
Rural area	729 (27.0%)	285 (29.2%)	124 (29.9%)	211 (24.0%)	54 (24.8%)
Missing	85 (3.1%)	4 (0.4%)	12 (2.9%)	30 (3.4%)	6 (2.8%)

Other outdoor workers (e.g. forester, gardener, construction worker), indoor workers (e.g. office worker, teacher), other occupations (e.g. both farmer and office worker); SD = standard deviation

### Self-reported prevalence of allergies

Of the participants, 27.3% indicated having at least 1 allergy. The most common elicitors were pollen (8.4%) and house dust mite (6.1%), followed by drug (5.4%), contact dermatitis (5.4%), and food (5.1%). The highest prevalence of allergies was observed in the age group 18–39 years (32.3%), while the age group 70+ years (20.5%) was least affected (p < 0.001).

The overall prevalence of any allergy was 19.9% in men and 33.7% in women (p < 0.001). In comparison to men, women showed a higher prevalence of all allergies, with contact (8.1% of women vs. 2.3% of men) and drug allergies (7.6% of women vs. 3.0% of men) showing the largest differences based on sex.

The lowest prevalence of allergies was found among famers (22.9%), whereas the highest prevalence was found among indoor workers (32.7%; p < 0.001). Pollen allergy was the most common allergy among other outdoor workers (8.2%), indoor workers (11.6%), and other occupations (12.5%). Only among farmers, contact allergies (5.3%) and drug allergies (5.0%) were more common than pollen allergy (4.6%; Table 2).

**Table 2:** Prevalence of self-reported allergies separated by age group, sex, occupational group and settlement structure.

	Any allergy	Pollen	House dust mite	Food	Contact allergy	Drug	Animal	Insect venom	Other
**Total**	27.3%	8.4%	6.1%	5.1%	5.4%	5.4%	3.4%	3.9%	1.3%
**Age group**									
18–39 years	32.3%	12.9%	9.6%	6.3%	3.5%	4.8%	5.5%	5.0%	0.9%
40–49 years	31.6%	8.6%	6.4%	5.7%	8.4%	6.2%	5.2%	4.4%	1.5%
50–59 years	27.9%	9.0%	5.7%	4.7%	6.4%	5.9%	2.8%	3.7%	1.2%
60–69 years	21.6%	4.7%	4.4%	4.6%	4.1%	5.5%	1.8%	3.0%	0.9%
70+ years	20.5%, p < 0.001	4.8%, p < 0.001	3.1%, p = 0.001	3.9%, p = 0.542	4.4%, p = 0.005	3.1%, p = 0.439	1.7%, p = 0.001	3.9% p = 0.513	1.7%, p = 0.744^1^
**Sex**									
Men	19.9%	6.2%	5.1%	3.0%	2.3%	3.0%	3.0%	3.4%	0.9%
Women	33.7%, p < 0.001	10.4%, p < 0.001	6.9%, p = 0.052	6.8%, p < 0.001	8.1%, p = 0.001	7.6%, p = 0.001	7.6%, p = 0.001	4.4%, p = 0.217	1.6%, p = 0.121
**Occupational group**									
Famers	22.9%	4.6%	4.5%	3.3%	5.3%	5.0%	2.2%	3.2%	1.5%
Other outdoor workers	23.1%	8.2%	6.0%	4.0%	4.7%	3.0%	3.7%	3.0%	1.2%
Indoor workers	32.7%	11.6%	8.4%	7.0%	5.4%	6.8%	4.8%	4.3%	1.1%
Other occupations	31.3%, p < 0.001	12.5%, p < 0.001	5.8%, p = 0.007	6.7%, p = 0.002	5.3%, p = 0.968	5.3%, p = 0.043	3.8%, p = 0.032	6.7%, p = 0.069	0.5%, p = 0.707^1^
**Settlement structure**									
Urban area	42.4%	18.4%	12.7%	9.5%	7.0%	7.6%	8.9%	6.3%	1.3%
Suburban area	27.1%	8.3%	6.0%	4.8%	5.5%	5.3%	3.3%	4.0%	1.1%
Rural area	24.4%, p < 0.001	6.1%, p < 0.001	5.4%, p = 0.002	4.5%, p = 0.029	4.9%, p = 0.584	5.2%, p = 0.451	3.0%, p = 0.001	3.5%, p = 0.268	1.4%, p = 0.746^1^

Other outdoor workers (e.g. forester, gardener, construction worker), indoor workers (e.g. office worker, teacher), other occupations (e.g. both farmer and office worker); ^1^ = exact Fisher test

**Fig. 1:**
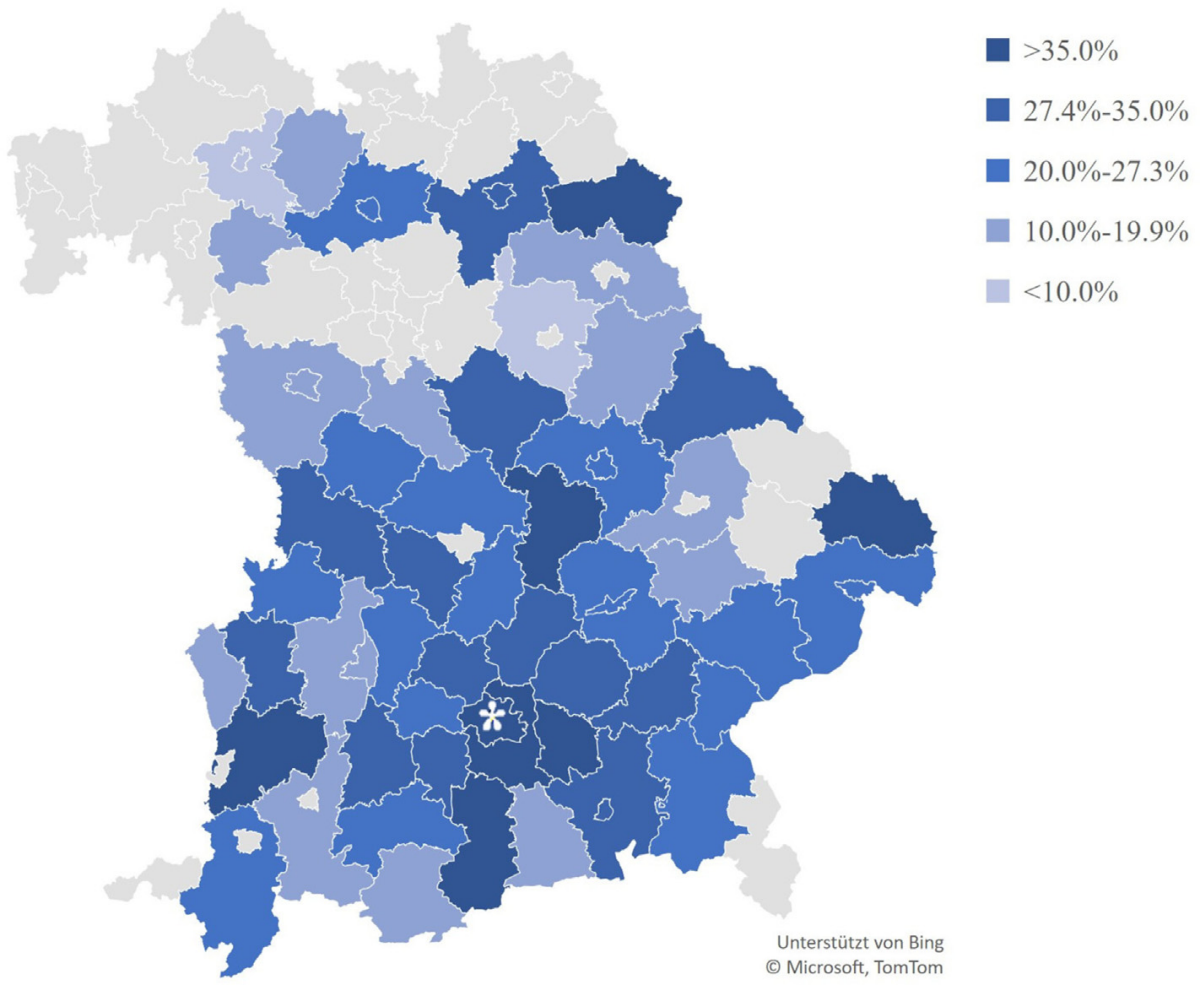
Proportion of individuals with self-reported allergy in different areas (n ≥ 15) in Bavaria, Southern Germany. ∗Munich

The highest self-reported prevalence of allergies was found in people living in urban areas (42.4%). Considering different Bavarian areas, the highest allergy rate was observed in Munich (40.1%, Fig. 1). Farmers living in suburban areas reported being more frequently affected by allergies (24.0%) than farmers living in rural areas did (18.7%). When excluding farmers, the prevalence of allergies was 47.2% in urban areas, 28.4% in suburban areas, and 27.7% in rural areas. Among indoor workers, the highest prevalence was observed in urban areas (48.5%), followed by rural areas (32.3%) and suburban areas (28.9%, Fig. 1).

### Multivariate logistic regression

Compared to the age group 70+ years, the age groups 18-39-years (odds ratio (OR) = 1.73; 95% CI [1.14; 2.65]) and 40-49-years (OR = 1.87; 95% CI [1.21; 2.89]) had a higher risk for having at least 1 allergy. Furthermore, women had a nearly 2 times higher risk for being affected by any allergy than men (OR = 1.82; 95% CI [1.50; 2.22]). Considering different kinds of allergies, women compared to men had a higher risk for contact (OR = 3.72; 95% CI [2.35; 5.91]), animal (OR = 2.15; 95% CI [1.30; 3.56]), and food allergy (OR = 2.07; 95% CI [1.35; 3.18]).

In comparison to farmers, the group of other occupations had the highest risk for pollen allergy (OR = 2.45; 95% CI [1.43; 4.17]). A significantly higher risk was also found among other outdoor workers (OR = 1.69; 95% CI [1.04; 2.76]) and among indoor workers (OR = 1.58; 95% CI [1.04; 2.41]). Furthermore, the groups of other occupations (OR = 2.02; 95% CI [1.03; 3.96]) and indoor workers (OR = 1.67; 95% CI [1.04; 2.69]) were more likely than farmers to be affected by food allergies. In general, people living in suburban areas (OR = 0.44; 95% CI [0.30; 0.64]) and rural areas (OR = 0.38; 95% CI [0.26; 0.58]) were less likely to report an allergy than people living in urban areas (Table 3).

**Table 3:** Results of the multivariate regression to assess risk factors for allergies in general and some specific allergies.

	Any allergy OR (95% CI)	Pollen OR (95% CI)	House dust mite OR (95% CI)	Food OR (95% CI)	Contact allergy OR (95% CI)	Animals OR (95% CI)
**Age group**						
18–39 years	**1.73 (1.14**–**2.65)**	**3.00 (1.32**–**6.80)**	**3.49 (1.47**–**8.28)**	–	0.49 (0.20–1.19)	2.12 (0.72–6.21)
40–49 years	**1.87 (1.21**–**2.89)**	2.18 (0.93–5.08)	2.04 (0.81–5.09)	–	1.51 (0.67–3.40)	2.17 (0.72–6.52)
50–59 years	1.47 (0.98–2.22)	2.11 (0.94–4.76)	1.87 (0.78–4.50)	–	1.15 (0.53–2.52)	1.15 (0.38–3.46)
60–69 years	1.12 (0.73–1.71)	1.20 (0.51–2.82)	1.49 (0.60–3.67)	–	0.74 (0.32–1.70)	0.84 (0.26–2.69)
70+ years	1	1	1	–	1	1
**Sex**						
Men	1	1	–	1	1	1
Women	**1.82 (1.50**–**2.22)**	**1.45 (1.04**–**2.02)**	–	**2.07 (1.35**–**3.18)**	**3.72 (2.35**–**5.91)**	**2.15 (1.30**–**3.56)**
**Occupational group**						
Famers	–	1	–	1	–	–
Other outdoor workers	–	**1.69 (1.04**–**2.76)**	–	1.24 (0.66–2.33)	–	–
Indoor workers	–	**1.58 (1.04**–**2.41)**	–	**1.67 (1.04**–**2.69)**	–	–
Other occupations	–	**2.45 (1.43**–**4.17)**	–	**2.02 (1.03**–**3.96)**	–	–
**Settlement structure**						
Urban area	1	1	1	1	–	1
Suburban area	**0.44 (0.30**–**0.64)**	**0.44 (0.27**–**0.73)**	**0.45 (0.26**–**0.78)**	**0.50 (0.27**–**0.95)**	–	**0.34 (0.18**–**0.66)**
Rural area	**0.38 (0.26**–**0.58)**	**0.31 (0.17**–**0.55)**	**0.39 (0.21**–**0.73)**	**0.45 (0.22**–**0.92)**	–	**0.30 (0.14**–**0.64)**

Other outdoor workers (e.g. forester, gardener, construction worker), indoor workers (e.g. office worker, teacher), other occupations (e.g. both farmer and office worker); CI = confidence interval; OR = Odds Ratio; bold printed indicates significance

### Treatment of allergies

**Fig. 2:**
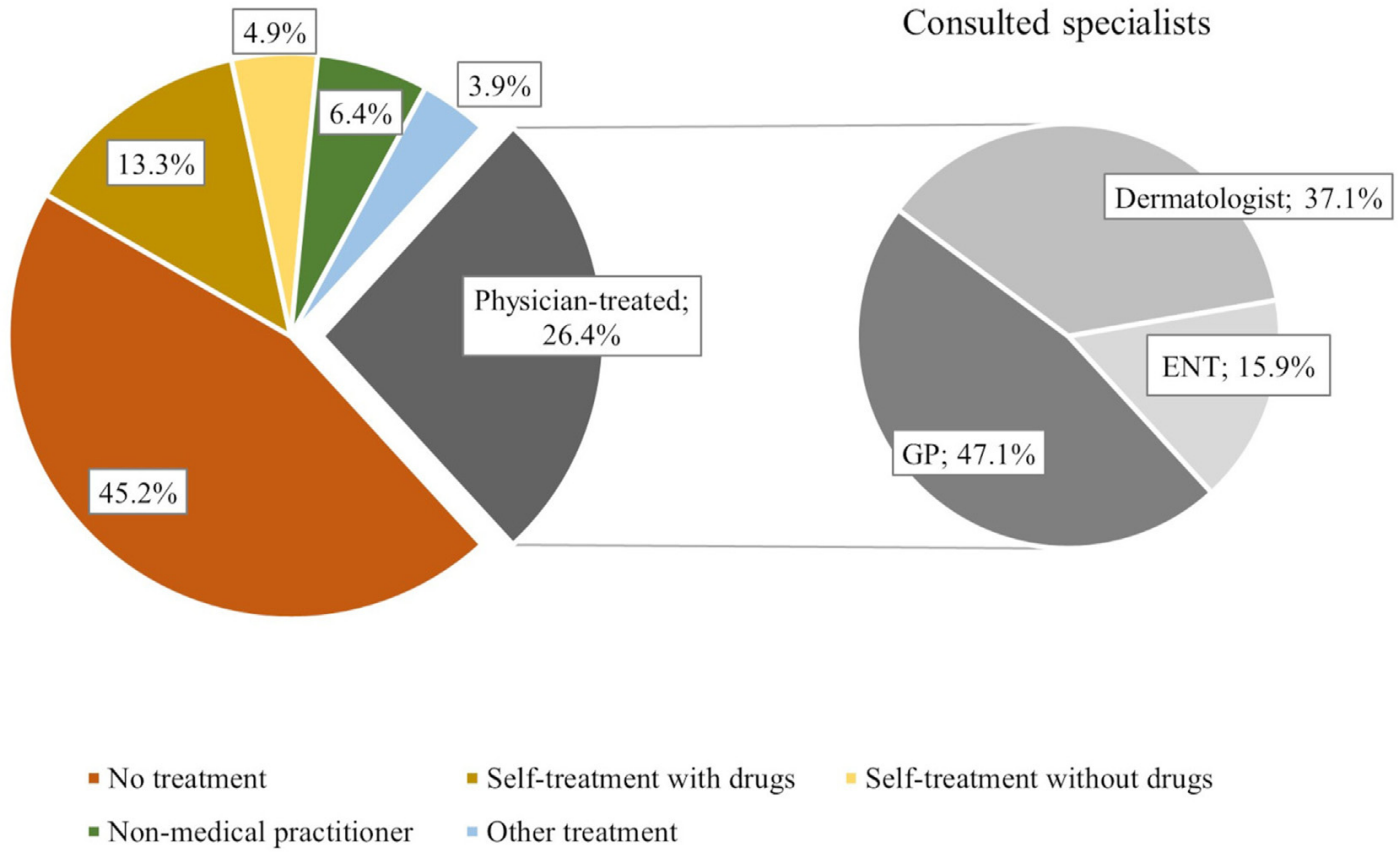
Proportion of treatment options used by people reported to be affected by an allergy (n = 675). ENT = ear nose throat specialist; GP = General practitioner

Of all participants with allergies (n = 712), a high number of individuals (45.2%) stated that their allergy was untreated. Around 26.4% reported being treated by a physician, with 47.1% of those being treated by a GP. However, 13.3% also reported that they treated their allergy themselves with drugs (Fig. 2). A comparison between women and men revealed that women were less likely to receive no treatment (OR = 0.58; 95% CI [0.41; 0.81]) and were more likely to consult a dermatologist (OR = 2.58; 95% CI [1.26; 5.29]) or non-medical practitioner (OR = 4.36; 95% CI [1.69; 11.25]).

## Discussion

The aim of the study was to assess the prevalence of allergies associated with individual characteristics like age, sex, occupation, and place of residence in a study population recruited outside a typical medical setting. Younger people, women, people living in urban areas, and indoor workers reported the highest prevalence of allergies.

The overall prevalence of self-reported allergies was 27.3%, which was similar to findings from a previous national study in Germany, which proposed a 12-month prevalence of 28.1%.^2^ Contrary to a study examining the allergy prevalence in rural areas of Bavaria, however, the detected prevalence was considerable lower (24.4%) than reported before (37.3%).^3^ Considering specific allergies, it was found that the prevalence of pollen allergy was markedly lower (14.8%), whereas the detected prevalence of contact allergy and insect venom allergy was higher in this study than reported in the literature (8.1% and 2.8%, respectively). These differences may be due to different recruitment methods and study population's characteristics such as a large proportion of outdoor workers in this study.^3^^,^^7^

Considering the occupational groups, great differences in the self-reported prevalence of pollen allergy were observed, whereby farmers were significantly less affected than all other occupational groups. While there is currently little information on the prevalence of pollen allergy in farmers, there is evidence that the sensitization to aeroallergens is lower among farmers than in non-farmers.^23^ A lower prevalence might be due to the fact that growing up on a farm lowers the risk of developing allergies even in adulthood.^11^^,^^12^^,^^24^, ^25^, ^26^ Another reason might be the consumption of raw milk on dairy farms, whose components have a protective effect through immunological processes.^27^, ^28^, ^29^ On dairy farms, breathing air with increased bacterial components in childhood can also decrease the reactivity of the immune system, which can prevent allergies diseases.^13^ Furthermore, the composition of the microbiome of farms could have a protective effect.^30^ As not all farmers spent their childhoods on farms, it warrants further investigation to determine if the exposure in childhood, the exposure at work, or both are responsible for the lower prevalence of pollen allergy. Questions on the exposure during early childhood were not included in the questionnaire and therefore could not be analysed in this study.

In addition to occupation, age, sex, and the residential area were also found to be associated with allergies. Like a previous German study, this study indicated that younger age groups had a higher risk for a few allergies such as pollen allergy;^7^ however, the study did not find that younger people are at a higher risk for food allergies.^31^ A reason for this could be that only people aged 18 years and older were included, in whom food allergy is already less common than in children. Regarding sex, it was found that women reported being affected by allergies more common. Comparable to the literature, in this study, the reported prevalence of a food allergy was twice as high in women than men and the reported prevalence of contact allergy was four times higher.^2^^,^^3^^,^^7^ One explanation for the higher prevalence of allergies in women is the influence of sex hormones on the development of allergies,^8^ but the higher self-reported prevalence might also be to some extend due to the general larger health awareness of women. In contrast to the literature, however, this study did not find that any occupational group had a higher risk for a contact allergy, which might be explained due to the low proportion of high risk occupations such as health care workers among indoor workers.^32^ Similar to previous studies, the study demonstrated a lower sensitization rate in rural areas than urban areas.^26^^,^^33^^,^^34^ A reason might be the higher exposure to traffic in urban areas, which is associated with allergic diseases.^35^^,^^36^ The observed lower prevalence of allergies in rural areas could be related to how lifestyles influence the development of allergies.^37^ For example, a change of lifestyle may explain the rapid increase in the prevalence of allergies observed in East Germany after German reunification.^38^, ^39^, ^40^ The current literature also highlights the connection between the settlement structure in which one was raised and sensitizations in adulthood. Adults who grew up in rural areas showed a significant risk reduction in being sensitized than adults who grew up in urban areas.^33^ As growing up with siblings was shown to coincide with a lower prevalence in allergies,^11^^,^^26^ growing up in a rural environment may provide a similar protective function because of the on average higher number of children in rural families.^41^ People living in urban areas having better access to medical specialists and thus consulting them more often than people living in rural areas is another possible explanation for the higher self-reported prevalence of allergies for urban citizens.^22^^,^^42^

In this study, the proportion of people whose allergy was not treated (45.2%) and the proportion of people whose allergy was treated by a physician (26.4%) were substantially higher and lower than the corresponding proportions in a previous study, respectively (30.4% and 64.1%).^3^ A reason for that might be that this study was based on data taken outside a medical setting. These findings highlight the continuous lack of sufficient allergy treatment for affected individuals. The benefits of adequate therapies, for example with AIT, can significantly lower the individual impairment of allergies while preventing the development of allergic asthma and other allergic comorbidities,^18^^,^^43^ which in turn can lower the overall global burden of allergies.^44^

There are some study limitations. One limitation is the possible selection bias due to the unconventional setting at the ZLF. Most participants in this setting were probably interested in agriculture and in allergies. As a self-administered questionnaire was used, a recall bias is also possible. Additionally, the reported prevalence was based on self-disclosure by the participants and was not confirmed with medical examinations. It is therefore possible that a higher prevalence was observed due to incorrectly reported information. Furthermore, urban areas were underrepresented compared to suburban areas and rural areas.

Apart from these limitations, the study provides a comprehensive overview of the prevalence of allergies in a large sample size that was recruited in an unconventional setting. Prevalence differences were observed for sex, age, and settlement structure as well as various occupations. Further studies should examine how childhood exposure influences the prevalence of pollen allergy among farmers. As almost half of the participants with an allergy did not receive treatment, future investigations should also find solutions to improving allergy therapies. By highlighting high-risk groups like indoor workers from urban areas and the treatment gap in allergies, our study seeks to raise the awareness of physicians regarding this matter, so that treatment can be adjusted adequately.

## Funding information

Parts of this study were financially supported by 10.13039/100008792Novartis Pharma GmbH and Beiersdorf Dermo Medical GmbH.

## Ethical disclosure

The study was reviewed and approved by the ethics review board of the Medical Faculty of Technical University of Munich (Reference 385/16 s) and all participants provided written informed consent prior to participation.

## Authors’ contribution

Conception or design of the work: Ring J., Eyerich K., Biedermann T., Zink A.

Acquisition of data: Tizek L., Biedermann T., Zink A.

Analysis or interpretation of data: Redlinger E., Tizek L., Ring J., Eyerich K., Zink A.

Drafting the work: Redlinger E., Tizek L., Zink A.

Revising it critically for important intellectual content: Tizek L., Ring J., Eyerich K., Biedermann T., Zink A.

Final approval of the version to be published: Redlinger E., Tizek L., Ring J., Eyerich K., Biedermann T., Zink A.

All authors read and approved the final manuscript.

## Submission declaration

This manuscript has not been published or presented elsewhere in part or in entirety and will not be submitted for publication elsewhere before a decision is reached concerning publication in WAO Journal. All authors approve the publication of this work.

## Availability of data and materials

The datasets used and/or analysed during the current study are available from the corresponding author on reasonable request.

## Declaration of competing interest

L. Tizek declares a conflict of interest with Novartis Pharma GmbH and Beiersdorf Dermo Medical GmbH.

E. Redlinger: none declared.

J. Ring: none declared.

Dr. Eyerich reports other from Novartis Pharma GmbH, other from Beiersdorf Dermo Medical GmbH, during the conduct of the study; grants and personal fees from Abbvie, personal fees from Almirall, personal fees from BMS, grants and personal fees from LEO, personal fees from Lilly, grants and personal fees from Janssen, grants and personal fees from UCB, grants and personal fees from Novartis, personal fees from Boehringer Ingelheim, outside the submitted work.

T. Biedermann gave advice to or got a honorarium for talks or research grants from the following companies: Alk-Abelló, Celgene-BMS, Lilly Deutschland GmbH, Mylan, Novartis Pharma GmbH, Phadia-Thermo Fisher, Sanofi-Genzyme, and Regeneron.

A. Zink has been an advisor and/or received speaker's honoraria and/or received unrestricted research grants and/or participated in clinical trials of the following companies: Abbvie, Amgen, Almirall, Beiersdorf Dermo Medical GmbH, Bencard Allergie, BMS, Celgene, Eli Lilly, GSK, Janssen Cilag, Leo Pharma, Miltenyi BiotecMiltenyi Biotec, Pfizer, Novartis, Phadia Thermofischer GmbH, Sanofi-Aventis, and Takeda Pharma.
